# Biofilm inhibition and pathogenicity attenuation in bacteria by *Proteus mirabilis*

**DOI:** 10.1098/rsos.170702

**Published:** 2018-04-18

**Authors:** Shichen Yu, Xiaoshan Zhu, Jin Zhou, Zhonghua Cai

**Affiliations:** 1 Shenzhen Public Service Platforms of Marine Microbial Resource Screening and Exploitation, Graduate School at Shenzhen, Tsinghua University, Shenzhen 518055, Guangdong, People's Republic of China; 2 School of Life Science, Tsinghua University, Beijing 100084, People's Republic of China

**Keywords:** biofilm, antibiotic resistance, quorum-sensing inhibitor, *Proteus mirabilis*

## Abstract

Biofilms play an important role in the antibiotic resistance of encased bacteria, and biofilm formation is regulated by quorum sensing (QS). Inhibiting the QS system may, therefore, degrade the integrity of a biofilm and expose the bacterial pathogens within it to the deleterious effects of molecules such as antibiotics. Moreover, the use of QS inhibitors (QSIs) may provide a novel approach for treating bacterial infections of aquacultures. In the present study, the bacterium *Proteus mirabilis* was identified as a potential producer of QSIs. Varying concentrations (0.1–1.1%) of filtrates prepared from the culture of *P. mirabilis* inhibited biofilm formation by the pathogens *Pseudomonas aeruginosa*, *Vibrio harveyi* and *Staphylococcus aureus* by as much as 58.9%, 41.5% and 41.9%, respectively. These filtrates as well as the crude aqueous extracts prepared from them increased the sensitivities of pathogens to the inhibitory effects of kanamycin. The filtrates also showed pathogenicity attenuation potential in *P. aeruginosa* by decreasing the production of virulence factors. Moreover, the filtrates did not influence the planktonic growth of these pathogens. The results indicate that *P. mirabilis* may act as a non-specific (or broad-spectrum) inhibitor of biofilm formation that will help control infectious diseases that adversely affect the aquaculture industry.

## 1. Introduction

Antibiotic resistance of pathogens is a global healthcare problem [1]. Overuse and abuse of antibiotics are the most important driving forces in the development of antibiotic resistance by pathogenic bacteria [2]. More than 100 000 tons of antibiotics are used by culture industries [3]. Industrial aquaculture is the fastest growing industry worldwide, and China is the largest aquaculture producer [4,5]. Thus, there is a growing urgency to search for a new therapeutic strategy for controlling aquatic infectious diseases.

Studies have demonstrated that bacterial biofilm is a potential contributor for the antibiotic resistance of pathogens [6]. The formation of biofilms by bacteria can impart as much as a 1000-fold increase in resistance to antimicrobials [7]. Bacterial biofilms, an extracellular matrix consisting of DNA, proteins and polysaccharides, are constructed by bacterial communities to form complex structures and adhere to living or non-living surfaces [8]. The polymeric matrix of the biofilm can retard the diffusion of antibiotics, making it more difficult for the antibiotics to penetrate the biofilm to eliminate the pathogens [9]. The protective architecture of the bacterial biofilm allows the survival of bacteria in hostile environments [10]. Therefore, the application of approaches for controlling biofilm formation for therapeutic approaches is highly desirable.

In the bacterial physiological process, one of the target regulatory mechanisms is quorum sensing (QS). It modulates (or controls) many phenotypes, such as biofilm formation, antibiotic production and virulence factor expression [11]. Interfering with the QS system may inhibit and eventually disrupt biofilm occurrence, thereby lessening the protection of biofilm in pathogens to deleterious agents, including antibiotics. The QS system and the associated QS inhibitors (QSIs) may, therefore, provide novel approaches for treating bacterial infections [12,13]. In fact, numerous QSIs have been identified in macroalgae, plants, fungi and actinomycetes; the compounds include halogenated furanone C30 and garlic extracts [13]. QSIs inhibit bacterial biofilm formation, rendering bacteria more susceptible to antibiotics [14]. Interestingly, unlike conventional antibiotics, QSIs only aid in the clearance of pathogens by destroying biofilm-mediated protection without influencing their growth or the selective pressures against them [12]. Moreover, QSIs may minimize the production of bacterial virulence factors and reduce the risk of septic shock during infection [12]. Thus, biofilm screening based on QSI seems a prudent and wise strategy to provide new therapies for bacterial infections and for controlling antibiotic resistance.

In the present study, QSI screening was performed to identify bacteria that might produce inhibitors of biofilm formation by typical pathogens such as *Pseudomonas aeruginosa*, *Vibrio harveyi* and *Staphylococcus aureus*. The potential of the inhibitor in attenuating the virulence of *P. aeruginosa* was also evaluated.

## 2. Material and methods

### 2.1. Screening of quorum sensing inhibitor-producing strains

Environmental microorganism samples were collected from a pond located on the campus of the Shenzhen Graduate School of Tsinghua University, Shenzhen, China. To enrich the bacteria with those that have colonizing ability, plastic plates were placed for 2 weeks in the pond and the samples were scraped from each plate and suspended individually in sterile phosphate-buffered saline. Single colonies were obtained by coating 10 µl of serially diluted suspensions on Luria-Bertani (LB) medium (yeast extract 5 g, peptone 10 g and NaCl 10 g in a final volume of 1000 ml of distilled water (pH 7.0), solidified with agar). To identify QSI-producing bacteria, the isolates were subjected to the QSI-screening procedure described below.

Screening the bacteria for QSI production was performed as described previously [15]. Briefly, the candidates were screened using *Chromobacterium violaceum* ATCC12472 as the reporter strain. Bacterial isolates that inhibited purple pigment formation by this reporter strain were considered potential candidates [16]. A second reporter strain, *Agrobacterium tumefaciens* A136 (TraI-lacZ fusion (pCF218) (pCF372), which produces a blue colour in the presence of 5-bromo-4-chloro-indolyl-β-d- galactopyranoside in response to AHL (acyl homoserine lactone, one kind of QS molecules), was used as the negative control [17]. *Pseudomonas aeruginosa* PAO-1 was used as a positive control. The two reporter strains, the positive control, and *V. harveyi* BB120 were all kindly provided by Dr Thomas Wood (The Pennsylvania State University, USA). Wild-type *S. aureus* was purchased from the China General Microbial Culture Collection (Beijing, China). All strains were cultured at 30°C in autoclaved LB broth medium.

### 2.2. Identification of quorum sensing inhibitor-producing bacteria

The potential QSI strains were grown overnight in LB broth at 30°C, and then a 200 µl aliquot of each culture was transferred into a clean 1.5 ml microfuge tube and centrifuged at 7000*g* for 1 min. The flow-through in the tube was discarded, 100 µl of TE buffer was added, and the sample was mixed gently and then boiled for 10 min. The resulting supernatant contained the DNA crude extract (OD_260_/OD_230_ was more than 1.7, and OD_260_/OD_280_ between 1.8 and 2.0). The 16S rRNA gene, which is approximately 1500 bp, was amplified by polymerase chain reaction using the forward primer 27F (5′-AGAGTTTGATCCTGGCTCAG-3′) and the reverse primer 1492R (5′-GGTTACCTTGTTACGACTT-3′) [18], and sequenced at BGI-Shenzhen (BGI China, Mainland). The sequences obtained were assembled, analysed and manually edited using a CAP3 software package. The resulting sequences were compared against those from the NCBI database (http://www.ncbi.nlm.nih.gov) using BLAST analysis. To further know the bacterial biochemical characters, the isolates were also analysed using a physico-chemical identification kit (Lichen Biological, Shanghai, China).

### 2.3. Crude extracts of the positive quorum sensing inhibitor strains

The potential QSI strain cultures were extracted according to a published method [12]. Briefly, the target strain was inoculated into 2 l of LB broth and incubated at 30°C on a rotary shaker for 2 days. The culture was centrifuged at 12 000 r.p.m. for 15 min at 4°C. The supernatant was collected and filtered through a 0.22 μm membrane. The filtrate was extracted twice using an equal volume of ethyl acetate. After extraction, the aqueous fraction was designated the aqueous crude extract, and the organic phase was designated the organic crude extract. The aqueous crude extract was concentrated using poly(ethylene glycol)-20000, and the organic crude extract was concentrated using a Rotavapor RII rotary evaporator (Buchi, Flawil, Switzerland).

### 2.4. Biofilm biomass assay

The effects of the QSI-positive strain culture filtrate on the biomass of biofilms produced by *P. aeruginosa* PAO1, *V. harveyi* BB120 and *S. aureus* were determined using the crystal violet (CV) method [19]. Briefly, freshly cultured bacteria were added to 96-well polystyrene plates (100 µl per well) and incubated in M63 medium supplemented with 1 mM MgSO_4_, 0.2% glucose and 0.5% casamino acids [20]. Different volumes of the strain filtrate (0.1%–1.1% v/v) or extracts (1% v/v) were added. The mixtures were incubated at 30°C for 48 h. Planktonic cells and spent medium were removed from each culture. The remaining adherent cells were gently rinsed twice using deionized water. A 100 µl aliquot of CV solution (1% w/v) was added to each well for 30 min at room temperature. The excess dye was discarded, and the plates were washed gently and thoroughly using deionized water. The CV-stained cells were solubilized in dimethylsulfoxide, and the absorbance at 570 nm was determined using a Varioskan Flash enzyme-linked immunosorbent assay reader (Thermo Scientific, Pittsburgh, PA, USA). *P. aeruginosa* PAO1, *V. harveyi* BB120 and *S. aureus* cultures incubated in the absence of the target strain filtrate served as negative controls. The QSI (*Z*)-4-bromo-5-(bromomethylene)-2(5H)-furanone (#53796; Sigma-Aldrich, St Louis, MO, USA) was used as a positive-control QSI. Experiments were performed with 12 replicates (12 replicate wells in 96-well plates) for each treatment. When absorbance was determined, three readings were recorded for each well. The experiments were repeated three times.

### 2.5. Biofilm structure determination

*Pseudomonas aeruginosa* PAO1, *V. harveyi* BB120 and *S. aureus* were cultured in M63 medium on a rotary shaker at 30°C with a sterilized coverslip placed in the media as a substrate for biofilm formation. A 1% volume of the target strain culture filtrate or crude aqueous extract was added, and the cultures were incubated for 3 days [21]. The biofilms were observed using scanning confocal laser microscopy (SCLM) with a model FV1000 microscope (Olympus, Tokyo, Japan). The thickness of a biofilm was determined by calculating the base of the biofilm cell cluster at the substratum and the apex of the biofilm cell cluster at the bulk–liquid interface farthest from the substratum.

### 2.6. Antibiotic sensitivity

*Pseudomonas aeruginosa* PAO1, *V. harveyi* BB120 and *S. aureus* were each cultured in the presence or absence of the filtrate/crude aqueous extract of cultured QSI strain in M63 medium for 3 days as described in the previous section. SCLM was used to measure the tolerance of these pathogenic bacteria in biofilms to antibiotics following treatment with QSI extracts. The antibiotic sensitivities of the pathogens were tested by adding kanamycin (kanamycin sulfate, a kind of aminoglycoside bactericidal antibiotic, final concentration 100 µg ml^−1^) to these cultures individually. One day later, the biofilm structure in all the experimental groups was determined, as mentioned above, using SCLM. In addition, the bacterial viability was determined using a LIVE/DEAD^®^ BacLight™ Bacterial Viability Kit (L-7012; Molecular Probes, Eugene, OR, USA) according to the Product Introduction and checked by SCLM [22]. This kit uses mixtures of SYTO 9 green fluorescent nucleic acid stain and the red fluorescent nucleic acid stain propidium iodide. These stains differ both in their spectral characteristics and in their ability to penetrate healthy bacterial cells. With an appropriate mixture of the SYTO 9 and propidium iodide stains, bacteria with intact cell membranes (live bacteria) stain fluorescent green, whereas bacteria with damaged membranes (dead bacteria) stain fluorescent red.

### 2.7. Growth rate

The influence of the filtrate of cultured QSI strain on the planktonic growth rate of *P. aeruginosa* PAO1, *V. harveyi* BB120 and *S. aureus* was determined. The three pathogenic bacteria species were cultured with different quantities of the QSI filtrate at 30°C on a rotary shaker. The absorbance was measured every hour at 600 nm using the enzyme-linked immunosorbent assay reader.

### 2.8. Pathogenicity assay

Virulence factors produced by *P. aeruginosa*, such as elastase and siderophores, and hydrolytic ectoenzymes are indicators of pathogenicity [23]. Elastase activity was measured using the elastin Congo red assay, and siderophores were detected using the chrome azurol S assay [24,25]. Ectoenzyme activities were detected using the following fluorogenic substrates (Sigma-Aldrich): l-leucine-7-amido-4-methylcoumarin hydrochloride for aminopeptidase, 4-methylumbelliferyl (MUF)-β-d-glucoside for β-glucosidase, MUF-phosphate for alkaline phosphatase and MUF-oleate for lipase [26]. The pathogens cultured in the absence of the QSI filtrate served as negative controls, and furanone served as the positive control.

### 2.9. Statistical analysis

All the results were reported as mean ± standard error (s.e.). The statistical analyses were performed using the Statistical Package for the Social Sciences (SPSS) for Windows program (version 13.0, SPSS Inc., San Rafael, CA). One-way analysis of variance (one-way ANOVA) with Tukey's multiple comparisons was used to compare the differences among different treatments. Differences were considered statistically significant when *p* < 0.05.

## 3. Results

### 3.1. Screening and identifying quorum sensing inhibitor-producing bacteria

**Figure 1.**
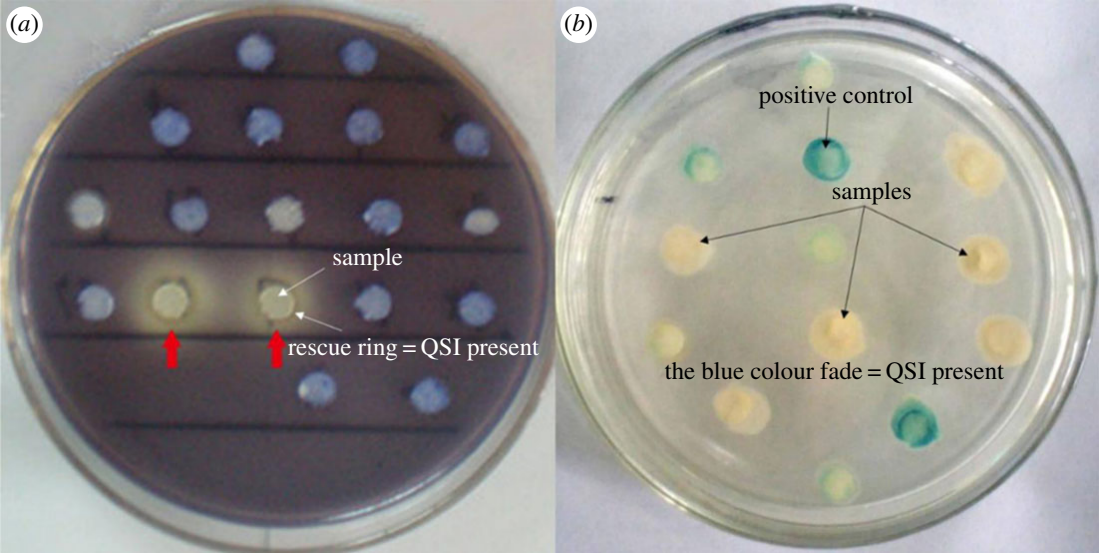
Screening of quorum-sensing inhibitors. (*a*) Screened by *C. violaceum* ATCC12472. Isolates with inhibition of purple pigment formation (rescue ring) were considered as QSI candidates. (*b*) Screened by *A. tumefaciens* A136. The fading of the blue colour indicated the presence of QSI with no interference of long-chain AHLs.

Hundreds of culturable bacteria were isolated from the plate. After screening the colonies, five isolates could inhibit QS-regulated purple pigment formation by *C. violaceum* (ATCC12472). These candidate isolates were further analysed using the *A. tumefaciens* A136 reporter strain to eliminate false positives produced by long-chain QS signals such as AHL (figure 1). This reporter strain produces a blue pigment in the presence of the long-chain AHL. Nucleotide sequence analysis revealed that the 16S rRNA genes of the five isolates were identical and shared greater than 99% identity with *Proteus mirabilis* strain NCTC 11938. The sequence was deposited in GenBank with the accession number NR 043997.1. The isolates tested positive for gelatin hydrolysis, urease, ornithine decarboxylase, xylopyranose and maltose fermentation, further confirming the identity of the isolates as *P. mirabilis* (table 1). *Proteus mirabilis* was stored at China General Microbiological Culture Collection (CGMCC no. 6426).

**Table 1.** Physiological and biochemical characterization of *P. mirabilis*.

characteristic	*P. mirabilis*
phenylalaninase	−
Simmon's citrate	−
gelatin hydrolysis	+
ornithine decarboxylase	+
urease	+
xylopyranose	+
maltose	+
hydrolysis of corn oil	−
d-mannitol	−
salicin	−
esculin	−

### 3.2. Inhibition of biofilm formation

**Figure 2.**
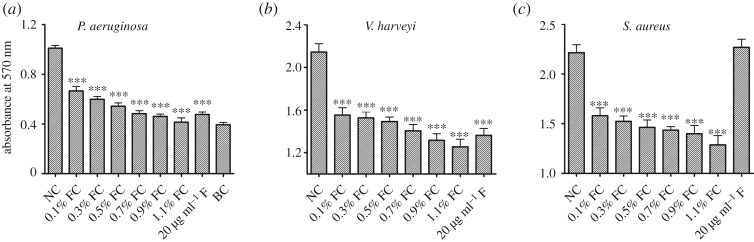
(*a*) Effect on *P. aeruginosa* PAO1. Samples were diluted 100-fold. (*b*) Effect on *V. harveyi* BB120. Samples were diluted 50-fold. (*c*) Effect on *S. aureus*. Samples were diluted 50-fold. Dilution ratios are for the samples, i.e. dimethylsulfoxide solution in which CV-stained cells were solubilized. The data represent the mean values of 12 replicates (12 wells in 96-well plates), with three readings for each replicate. Significant differences are indicated by asterisks (***p < 0.001).

The effects of filtrates prepared from the culture of *P. mirabilis* on the biomass of biofilms formed in cultures of three bacterial pathogens are shown in figure 2 (NC, negative control; FC, filtrate of cultured *P. mirabilis*; F, furanone (20 µg ml^−1^); BC, blank control). The filtrate (0.1–1.1%, v/v) effectively inhibited the production of biofilm biomass by each pathogen in a dose-dependent manner. The highest percentage inhibition of biofilm formation by each culture using a 1.1% concentration of the filtrate was as follows: 58.9%, 41.5% and 41.9% for *P. aeruginosa*, *V. harveyi* and *S. aureus*, respectively. The reference, 20 µg ml^−1^ furanone, was as effective as the 0.7% filtrate but had no effect on the biofilm biomass of *S. aureus*.

**Figure 3.**
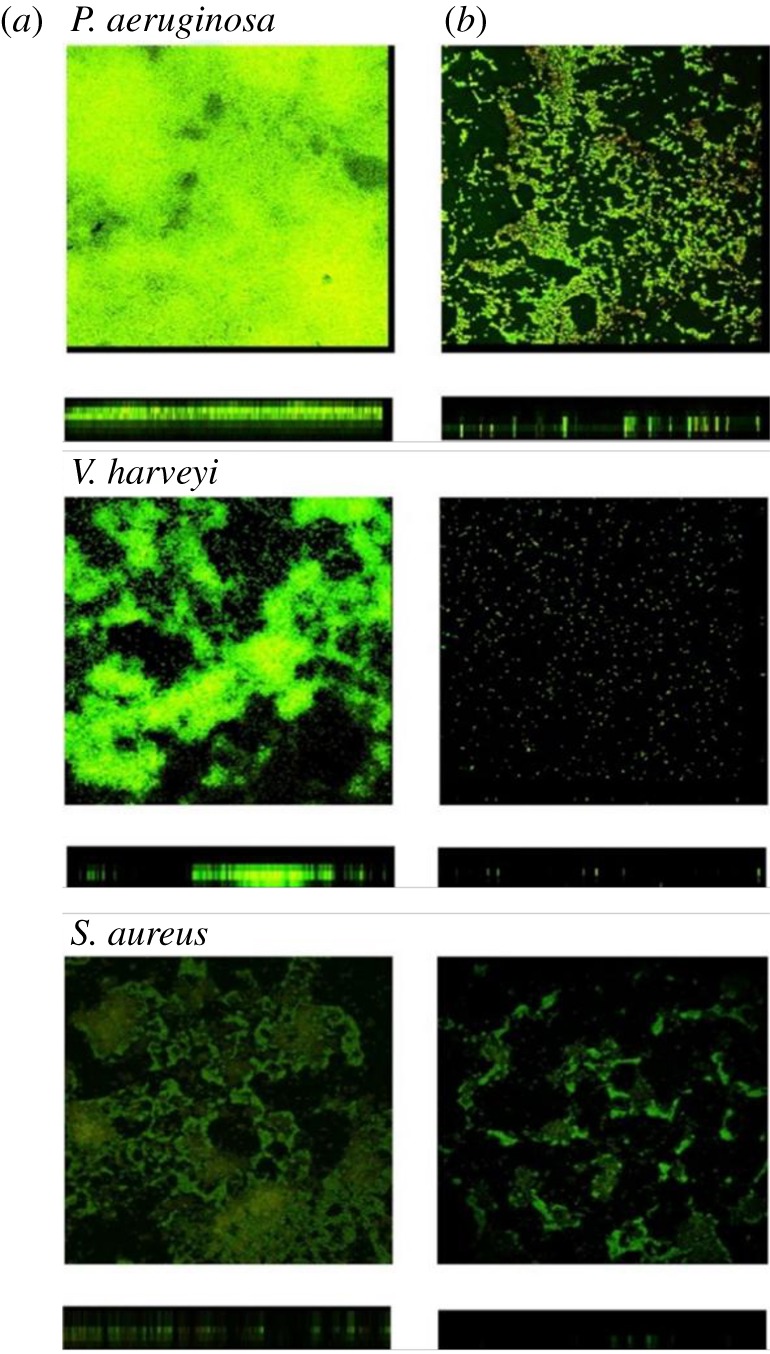
Biofilm structures of the three selected pathogenic bacteria after inhibition by the filtrate of *P. mirabilis*. Cultures of pathogenic bacteria were incubated for 3 days with the 1% (v/v) *P. mirabilis* filtrate. Red and green areas indicate dead and viable bacteria, respectively. Images were taken by SCLM. (*a*) With no filtrate; (*b*) with 1% (v/v) filtrate. *N* = 5, 10 × 100.

The inhibition of biofilm formation by the filtrate was further confirmed using SCLM observation. Pathogens formed biofilms on coverslips in M63 medium with or without the filtrate or a crude extract. After a 3-day incubation, the coverslip was washed and stained with bacterial viability dye. In contrast to the negative control, pathogen biofilms treated with a 1% filtrate of *P. mirabilis* cultures were thinner and less adherent (figure 3). In the presence of the 1% filtrate, the thickness of biofilms formed by *P. aeruginosa*, *V. harveyi* and *S. aureus* decreased from 9 to 5 µm, 10 to 6 µm and 5 to 3 µm, respectively (figure 3, table 2). *Vibrio harveyi* treated with the 1% filtrate did not form biofilms (figure 3). Taken together, these results demonstrate that *P. mirabilis* isolates, selected for their ability to inhibit QS, produced an inhibitor of biofilm formation and caused biofilm disruption.

**Table 2.** Thickness of biofilms treated with kanamycin and a filtrate prepared from cultures of *P. mirabilis*.

strain	negative control	1% filtrate of cultured *P. mirabilis*	100 µg ml^−1^ kanamycin	1% filtrate of cultured *P. mirabilis* and 100 µg ml^−1^ kanamycin
*P. aeruginosa*	9 µm ± 0.5	6 µm ± 0.5***	10 µm ± 1	5 µm ± 0.5***
*V. harveyi*	10 µm ± 2	6 µm ± 0.5***	9 µm ± 1	5 µm ± 0.5***
*S. aureus*	5 µm ± 0.5	3 µm ± 0.5**	5 µm ± 0.5	3 µm ± 0.5**

Significant differences are indicated by asterisks (**p < 0.01, ***p < 0.001).

### 3.3. Antibiotic sensitivity

**Figure 4.**
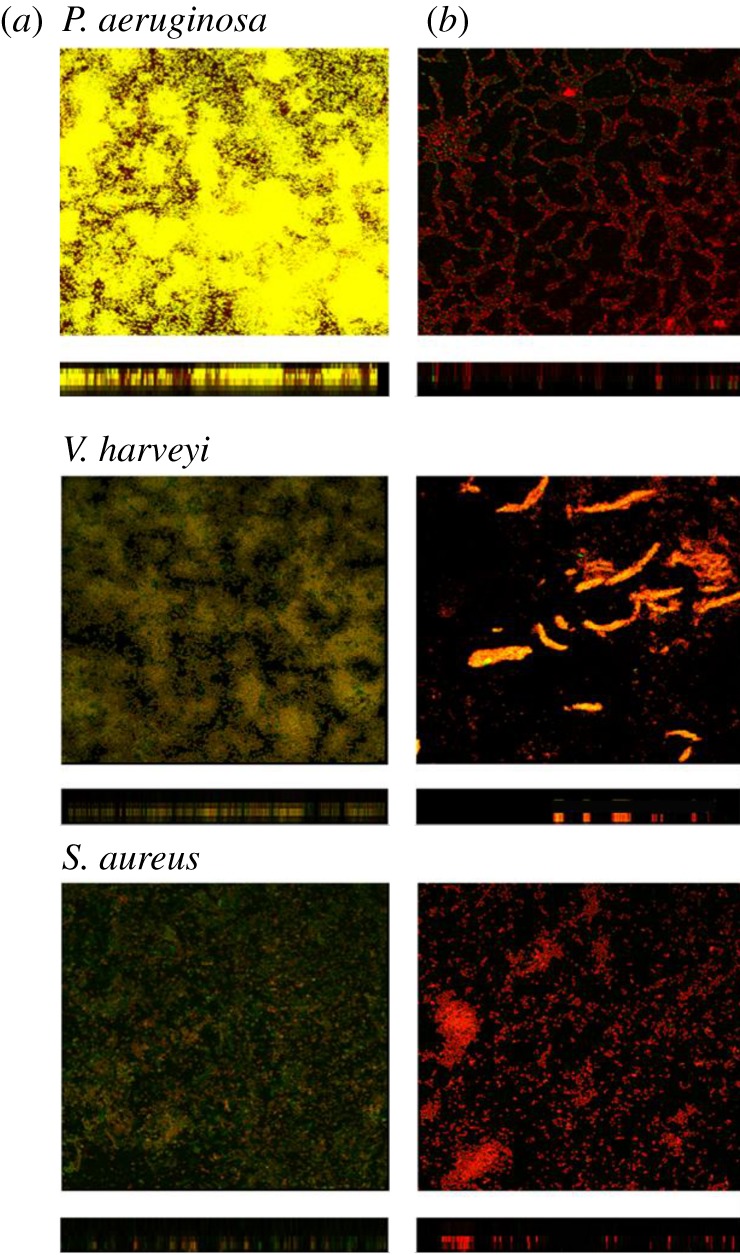
Effects of kanamycin and the filtrate of *P. mirabilis* on the biofilms formed by pathogenic bacteria. (*a*) Effect of 100 µg ml^−1^ kanamycin only. Biofilms formed by the pathogenic bacteria, after culturing for 3 days with no filtrate of *P. mirabilis* + 1 day 100 µg ml^−1^ kanamycin exposure. (*b*) Combined effects of kanamycin and the filtrate. Biofilms formed by the pathogenic bacteria, after culturing for 3 days with 1% (v/v) filtrate of *P. mirabilis* + 1 day 100 µg ml^−1^ kanamycin exposure. Red, green and yellow/brown areas indicate the dead bacteria, live bacteria, and the combined or overlapping of the dead and live bacteria, respectively. *N* = 5, 10 × 100.

The sensitivity of pathogens to antibiotics was assessed in the presence or absence of the *P. mirabilis* culture filtrate. Kanamycin alone did cause a decrease in the viability of each of the pathogens in the biofilms (figure 4). However, only the cells on or just below the surface of the biofilms were sensitive to kanamycin. Bacteria located deeper within the biofilms might still survive. When biofilms were treated with both the 1% filtrate and kanamycin, the sensitivity of each pathogen to the antibiotics increased significantly. Most of the cells were killed by 100 µg ml^−1^ kanamycin (figure 4).

### 3.4. Effect of the *P. mirabilis* culture filtrate on the planktonic growth of bacteria

**Figure 5.**
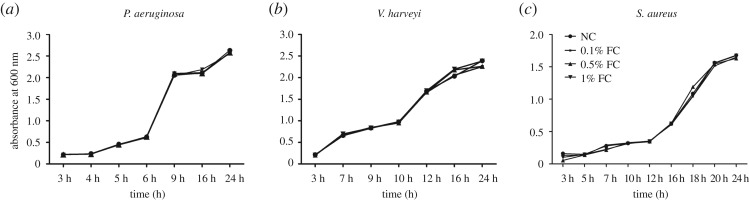
Influence of the *P. mirabilis* filtrate on bacterial growth. (*a*) *Pseudomonas aeruginosa*; (*b*) *V. harveyi*; (*c*) *S. auerus*. Cultures were incubated for 24 h in LB medium in the absence (circles) or presence of 0.1% (squares), 0.5% (triangles) or 1% (inverted triangles) concentrations of the *P. mirabilis* filtrate. NC, negative control; FC, filtrate of cultured *P. mirabilis*.

Growth curves were determined in the presence of 0.1%, 0.5% and 1% concentrations of the *P. mirabilis* culture filtrate. There was no significant difference among the experimental and control groups (figure 5).

### 3.5. Analysis of pathogenicity

**Figure 6.**
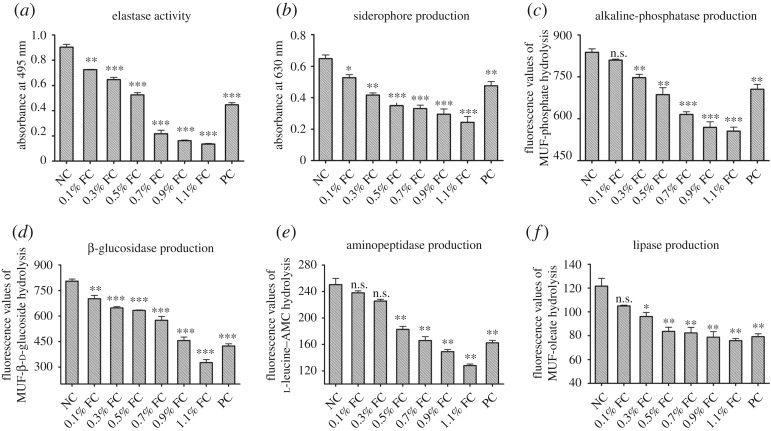
Attenuation of pathogenicity of *P. aeruginosa* by the filtrate of cultured *P. mirabilis*. (*a*) Elastase activity; (*b*) siderophore production; (*c*) alkaline phosphatase production; (*d*) β-glycosidase production; (*e*) aminopeptidase production; (*f*) lipase production. Cultures of *P. aeruginosa* were incubated in LB medium with or without the filtrate of cultured *P. mirabilis*. A well-known QSI (20 µg ml^−1^ furanone) was used as a positive control. NC, negative control; FC, filtrate of cultured *P. mirabilis*; PC, positive control (20 µg ml^−1^ furanone). *p < 0.05, **p < 0.01 and ***p < 0.001.

The influence of the filtrates prepared from the cultures of *P. mirabilis* on the virulence of *P. aeruginosa* is shown in figure 6. Elastase, siderophore and ectoenzymes were selected to indicate bacterial virulence using standard methods described in Material and methods. Elastase activities, siderophore production and ectoenzyme activities in the *P. aeruginosa* treated by filtrates of *P. mirabilis* culture were inhibited significantly compared with those of the negative control (figure 6*a–f*). The activities of alkaline phosphatase, β-glucosidase, aminopeptidase and lipase in the *P. aeruginosa* were all inhibited by the filtrate of cultured *P. mirabilis*.

### 3.6. Effects of two crude extracts of the *P. mirabilis* culture filtrate

The *P. mirabilis* culture filtrate was extracted using ethyl acetate, and the bioactivity of these extracts on biofilm biomass and virulence factors was determined. The crude aqueous extracts of *P. mirabilis* were stronger inhibitors of biofilm formation by *P. aeruginosa*, *V. harveyi* and *S. aureus* (table 3) as well as the activities of virulence factors (elastase activity, siderophore production and hydrolytic ectoenzyme activity) (table 4) than those of the organic extracts.

**Table 3.** Effect of *P. mirabilis* crude extracts on biofilm formation. AE, aqueous crude extracts of *P. mirabilis*; OE, organic crude extracts of *P. mirabilis*; ± s.d.

	biofilm biomass (absorbance at 570 nm)		
crude extracts	*P. aeruginosa*	*V. harveyi*	*S. aureus*
negative control	1.01 ± 0.06	2.14 ± 0.22	2.21 ± 0.23
1% AE	0.46 ± 0.09***	1.23 ± 0.13***	1.21 ± 0.23***
1% OE	0.73 ± 0.13***	2.03 ± 0.25	2.15 ± 0.24

Significant difference is indicated by ***p < 0.001.

**Table 4.** Effect of the crude extracts of *P. mirabilis* on virulence factors of *P. aeruginosa*. AE, aqueous crude extracts of *P. mirabilis*; OE, organic crude extracts of *P. mirabilis*; data are listed as mean ± s.e. The errors are from six replicate wells in 96-well plates, with three readings for each well.

crude extracts	elastase	siderophore	alkaline phosphatase	β-glucosidase	aminopeptidase	lipase
negative control	0.904 ± 0.039	0.648 ± 0.040	838.0 ± 21.5	805.4 ± 19.7	250.5 ± 16.1	84.2 ± 13.8
1% AE	0.144 ± 0.007***	0.332 ± 0.047**	777.4 ± 19.0**	532.4 ± 16.2***	181.2 ± 5.1**	103.1 ± 8.9*
1% OE	0.825 ± 0.006**	0.599 ± 0.049	813.1 ± 8.8	692.1 ± 13.1***	229.5 ± 13.9	121.6 ± 11.3**

Significant difference is indicated by *p < 0.05, **p < 0.01 and ***p < 0.001.

## 4. Discussion

The QS signalling system senses the density of bacterial populations and plays a pivotal role in bacterial communication and regulation of metabolism [27,28]. The expression of more than 6% of the genes of most infectious bacteria is regulated by the QS system, including genes encoding proteins involved in biofilm development, as well as by exotoxin A, catalase, siderophores and exoenzymes [29]. The disruption of a pathogen's QS system might prevent and control a number of bacterial diseases that affect the biotechnology and aquaculture industries [30].

In the present study, we screened for bacteria with QSI activity in freshwater using the reporter strains *C. violaceum* ATCC 12472 and *A. tumefaciens* A136. We succeeded in isolating *P. mirabilis*. The filtrate of the *P. mirabilis* cultures was found to significantly inhibit the biofilm formation of Gram-negative pathogens *P. aeruginosa* and *V. harveyi* and the Gram-positive pathogen *S. aureus* (figures 1 and 2, table 2). These two classes of bacterial pathogens employ the following different QS signalling systems: AHLs in Gram-negative and autoinducing peptides in Gram-positive bacteria [31]. This inhibition activity seems similar to that of halogenated furanone, which is one of the well-known QSIs. Furanone could inhibit biofilm formation and attenuate the virulence of *Escherichia coli* by interfering with its AI-2 QS system [22]. It was also found to disrupt biofilm formation in *Streptococcus* mutants by interfering with *luxS*-dependent QS signalling [32]. However, furanone did not change the biofilm formed by *S. aureus*, which is different from that observed in the filtrate of the *P. mirabilis* cultures (figure 2). These results indicate that the elected biofilm inhibitor in this study may be a non-specific antagonist of the QS signalling system, and have complex inhibition mechanisms. In addition, it is worth noting that previous study demonstrated dispersed cells were more virulent compared with biofilm planktonic cells [33]; hence, in future work, biofilm disruption and bacteria elimination need to be performed simultaneously in order to better understand the biofilm influence mechanisms [34].

To identify the bioactive compounds including QSIs, the filtrate of the culture was extracted with ethyl acetate, and the aqueous and organic phases were analysed for their effects on biofilm formation. Only the aqueous crude extracts suppressed biofilm formation (table 3). Moreover, bioactivity decreased quickly when samples were stored at 4°C (data not shown), indicating that the bioactive compounds produced by *P. mirabilis* might be enzymes, although more experiments need to be conducted to test it. Quorum-quenching enzymes purified from certain bacteria have been shown to inhibit biofilm formation [12]. In the present study, inhibition of the QS or biofilm system by bacterial extracts was observed, although the composition of the compounds responsible for this phenotype is unknown. We speculate there are two possible reasons: (1) multiple chemicals produced by the bacteria cause unique effects at a variety of points in the QS system or (2) these compounds are not directly acting on the AI-1 system but, instead, are interfering with a global regulator of QS such as AI-2 [35]. However, to completely understand these broad-spectrum inhibition effects, further studies are ongoing to investigate the mechanism of action of bioactive compounds in the filtrate of *P. mirabilis* culture.

Because of their complex architecture, biofilms are difficult for antibiotics or other inhibitory agents to penetrate, and therefore they increase the resistance of bacteria to antibiotics [7]. Residual pathogens in a biofilm may gradually adapt to antibiotic pressure and evolve a new resistance mechanism [13]. To explore the potential application of the biofilm inhibitor identified here for treating bacterial infections, we compared its activity to kanamycin and determined whether these agents synergized. The *P. mirabilis* culture filtrate did not inhibit the growth of the bacterial pathogens studied here, although it inhibited their biofilm formation. After culturing the three different pathogens with the *P. mirabilis* culture filtrate, most of the cells remained viable, but the biofilms were thin and not intact (figure 3). We further tested the antibiotic sensitivity of biofilms formed by these pathogens in the presence and absence of the *P. mirabilis* filtrate. At the same concentration of kanamycin, the antibiotic alone killed only bacteria located on the surface of the biofilm, whereas kanamycin applied together with the filtrate killed most of the bacteria residing in the biofilm. The susceptibility of the pathogens to antibiotics was significantly improved after the biofilms were destroyed (figures 3 and 4). These results suggest that the *P. mirabilis* culture filtrate synergized with kanamycin. More research works using other antibiotics from a different class with a different mode of action are warranted to further prove this synergistic activity of the *P. mirabilis* culture filtrate on the sensitivity of pathogens to antibiotics.

To cause infections, pathogens must enter their host and withstand host defence mechanisms [36]. To achieve this goal, bacteria synthesize virulence factors to damage host tissues [37]. The QS system of *P. aeruginosa* not only regulates biofilm formation but also triggers the expression of QS-regulated genes encoding virulence factors [38]. We tested the effects of the filtered *P. mirabilis* culture on the production of elastase, siderophores and several hydrolytic ectoenzymes produced by *P. aeruginosa*. The levels of each were significantly decreased in the presence of filtrates or crude aqueous extracts of *P. mirabilis* (figure 6 and table 4).

To summarize, we identified the *P. mirabilis* QSI-producing strain using an environmental screening method. Extracts of *P. mirabilis* were antagonistic to pathogenic bacteria QS and affected QS-regulated phenotypes, including biofilm formation, virulence factor production and antibiotic susceptibility. Interestingly, extracts of *P. mirabilis* did not affect the growth of *P. aeruginosa* PAO1. Our experiments represent a first step in isolating new and more effective inhibitors of biofilm formation that may prove to be important for protecting industrial aquaculture from the devastating effects of bacterial infections. However, the chemical compounds and the active mechanisms of the biological extract are needed for further identification, and these works are ongoing in our laboratory.

## Data accessibility

All data are included in the paper or appendices, or have been uploaded as part of the electronic material. Data are available from the Dryad Digital Repository: http://dx.doi.org/10.5061/dryad.sg4n7kb [39].

## References

[1] Becattini S, Taur Y, Pamer EG 2016 Antibiotic-induced changes in the intestinal microbiota and disease. Trends. Mol. Med. 22, 458–478. (doi:10.1016/j.molmed.2016.04.003) 27178527 10.1016/j.molmed.2016.04.003 PMC4885777

[2] Augustine N, Peter AW, Kerkar S, Thomas S. 2012 Arctic actinomycetes as potential inhibitors of *Vibrio cholerae* biofilm. Curr. Microbiol. 64, 1–5. (doi:10.1007/s00284-011-0073-4) 22231452 10.1007/s00284-011-0073-4

[3] Aarestrup FM 2000 Occurrence, selection and spread of resistance to antimicrobial agents used for growth promotion for food animals in Denmark. APMIS. Suppl. 101, 1–48. 11125553

[4] Bostock J et al. 2010 Aquaculture: global status and trends. Phil. Trans. R. Soc. B 365, 2897–2912. (doi:10.1098/rstb.2010.0170) 20713392 10.1098/rstb.2010.0170 PMC2935128

[5] Department FF. 2004 *The state of world fisheries and aquaculture*. Rome, Italy: Food and Agriculture Organization.

[6] Lewis DD, Ruane ML, Scheeline A 2006 Biofilm effects on the peroxidase-oxidase reaction. J. Phys. Chem. B 110, 8100–8104. (doi:10.1021/jp0565608) 16610912 10.1021/jp0565608

[7] Anwar H, Dasgupta M, Costerton J 1990 Testing the susceptibility of bacteria in biofilms to antibacterial agents. Antimicrob. Agents Chemother. 34, 2043 (doi:10.1128/AAC.34.11.2043) 2073094 10.1128/aac.34.11.2043 PMC171995

[8] O'Toole GA 2003 To build a biofilm. J. Bacteriol. 185, 2687–2689. (doi:10.1128/JB.185.9.2687-2689.2003) 12700246 10.1128/JB.185.9.2687-2689.2003 PMC154388

[9] Korukluoglu M, Gulgor G 2016 The correlation between biofilm formation and antibiotic resistance of some microorganisms isolated from ‘Kefir’. J. Biotechnol. 231, S15 (doi:10.1016/j.jbiotec.2016.05.077)

[10] Costerton JW, Stewart PS, Greenberg EP 1999 Bacterial biofilms: a common cause of persistent infections. Science 284, 1318–1322. (doi:10.1126/science.284.5418.1318) 10334980 10.1126/science.284.5418.1318

[11] Sethupathy S, Prasath KG, Ananthi S, Mahalingam S, Balan SY, Pandian SK 2016 Proteomic analysis reveals modulation of iron homeostasis and oxidative stress response in *Pseudomonas aeruginosa* PAO1 by curcumin inhibiting quorum sensing regulated virulence factors and biofilm production. J. Proteomics 145, 112–126. (doi:10.1016/j.jprot.2016.04.019) 27108548 10.1016/j.jprot.2016.04.019

[12] Dong YH, Zhang LH 2005 Quorum sensing and quorum-quenching enzymes. J. Microbiol. 43, 101–109. 15765063

[13] Kociolek MG 2010 Quorum-sensing inhibitors and biofilms. Anti-Infect. Agents Med. Chem. 8, 315–326. (doi:10.2174/187152109789760117)

[14] Sethupathy S, Shanmuganathan B, Kasi PD, Pandian SK 2016 Alpha-bisabolol from brown macroalga *Padina gymnospora* mitigates biofilm formation and quorum sensing controlled virulence factor production in *Serratia marcescens*. J. Appl. Phycol. 28, 1987–1996. (doi:10.1007/s10811-015-0717-z)

[15] McLean RJC, Pierson LS 2004 A simple screening protocol for the identification of quorum signal antagonists. J. Microbiol. Methods 58, 351–360. (doi:10.1016/j.mimet.2004.04.016) 15279939 10.1016/j.mimet.2004.04.016

[16] Sanchez GV, Master RN, Clark RB, Fyyaz M, Duvvuri P, Ekta G, Bordon J 2013 *Klebsiella pneumoniae* antimicrobial drug resistance, United States, 1998–2010. Emerg. Infect. Dis. 19, 133–136. (doi:10.3201/eid1901.120310) 23260464 10.3201/eid1901.120310 PMC3557979

[17] Fuqua C, Winans SC 1996 Conserved cis-acting promoter elements are required for density-dependent transcription of *Agrobacterium tumefaciens* conjugal transfer genes. J. Bacteriol. 178, 435–440. (doi:10.1128/jb.178.2.435-440.1996) 8550463 10.1128/jb.178.2.435-440.1996 PMC177675

[18] Weisburg WG, Barns SM, Pelletier DA, Lane DJ 1991 16S ribosomal DNA amplification for phylogenetic study. J. Bacteriol. 173, 697–703. (doi:10.1128/jb.173.2.697-703.1991) 1987160 10.1128/jb.173.2.697-703.1991 PMC207061

[19] Huber B, Eberl L, Feucht W, Polster J 2003 Influence of polyphenols on bacterial biofilm formation and quorum-sensing. Z. Naturfosch. C 58, 879–884. (doi:10.1515/znc-2003-11-1224) 10.1515/znc-2003-11-1224 14713169

[20] Hinsa SM 2006 Biofilm formation by *Pseudomonas fluorescens* WCS365: a role for LapD. Microbiology 52(Pt 5), 1375–1383. (doi:10.1099/mic.0.28696-0) 10.1099/mic.0.28696-0 16622054

[21] Colvin KM, Gordon VD, Murakami K, Borlee BR, Wozniak DJ, Wong GCL, Parsek MR 2011 The pel polysaccharide can serve a structural and protective role in the biofilm matrix of *Pseudomonas aeruginosa*. PLoS Pathog. 7, e1001264 (doi:10.1371/journal.ppat.1001264) 21298031 10.1371/journal.ppat.1001264 PMC3029257

[22] Ren D, Sims J, Wood T 2002 Inhibition of biofilm formation and swarming of *Bacillus subtilis* by (5Z)-4-bromo-5-(bromomethylene)-3-butyl-2 (5H)-furanone. Lett. Appl. Microbiol. 34, 293–299. (doi:10.1046/j.1472-765x.2002.01087.x) 11940163 10.1046/j.1472-765x.2002.01087.x

[23] Antunes LCM, Ferreira RB, Buckner MM, Finlay BB 2010 Quorum sensing in bacterial virulence. Microbiology 156(Pt 8), 2271–2282. (doi:10.1099/mic.0.038794-0) 20488878 10.1099/mic.0.038794-0

[24] Pearson JP 1997 Roles of *Pseudomonas aeruginosa las* and *rhl* quorum-sensing systems in control of elastase and rhamnolipid biosynthesis genes. J. Bacteriol. 179, 5756–5767. (doi:10.1128/jb.179.18.5756-5767.1997) 9294432 10.1128/jb.179.18.5756-5767.1997 PMC179464

[25] Schwyn B, Neilands J 1987 Universal chemical assay for the detection and determination of siderophores. Anal. Biochem. 160, 47–56. (doi:10.1016/0003-2697(87)90612-9) 2952030 10.1016/0003-2697(87)90612-9

[26] Hoppe H 2001 Significance of exoenzymatic activities in the ecology of brackish water: measurements by means of methylumbelliferyl-substrates. Mar. Ecol. Prog. Ser. 11, 299–308. (doi:10.3354/meps011299)

[27] Schauder S, Bassler BL 2001 The languages of bacteria. Genes Dev. 15, 1468–1480. (doi:10.1101/gad.899601) 11410527 10.1101/gad.899601

[28] Waters CM, Lu W, Rabinowitz JD, Bassler BL 2008 Quorum sensing controls biofilm formation in *Vibrio cholerae* through modulation of cyclic di-GMP levels and repression of *vpsT*. J. Bacteriol. 190, 2527–2536. (doi:10.1128/JB.01756-07) 18223081 10.1128/JB.01756-07 PMC2293178

[29] Adonizio A, Kong KF, Mathee K 2008 Inhibition of quorum sensing-controlled virulence factor production in *Pseudomonas aeruginosa* by South Florida plant extracts. Antimicrob. Agents Chemother. 52, 198–203. (doi:10.1128/AAC.00612-07) 17938186 10.1128/AAC.00612-07 PMC2223872

[30] Austin B, Zhang XH 2006 *Vibrio harveyi*: a significant pathogen of marine vertebrates and invertebrates. Lett. Appl. Microbiol. 43, 119–124. (doi:10.1111/j.1472-765X.2006.01989.x) 16869892 10.1111/j.1472-765X.2006.01989.x

[31] Miller MB, Bassler BL 2001 Quorum sensing in bacteria. Annu. Rev. Microbiol. 55, 165–199. (doi:10.1146/annurev.micro.55.1.165) 11544353 10.1146/annurev.micro.55.1.165

[32] Huang Z, Meric G, Liu Z, Ma R, Tang Z, Lejeune P 2009 *luxS*-based quorum-sensing signaling affects biofilm formation in *Streptococcus mutans*. J. Mol. Microbiol. Biotechnol. 17, 12–19. (doi:10.1159/000159193) 18818488 10.1159/000159193

[33] Sintim HO, Gürsoy UK 2016 Biofilms as ‘connectors’ for oral and systems medicine: a new opportunity for biomarkers, molecular targets, and bacterial eradication. OMICS 20, 3–11. (doi:10.1089/omi.2015.0146) 26583256 10.1089/omi.2015.0146 PMC4739346

[34] Chua SL et al. 2014 Dispersed cells represent a distinct stage in the transition from bacterial biofilm to planktonic lifestyles. Nat. Commun. 5, 4462 (doi:10.1038/ncomms5462) 25042103 10.1038/ncomms5462

[35] Reimmann C et al. 1997 The global activator GacA of *Pseudomonas aeruginosa* PAO positively controls the production of the autoinducer N-butyryl- homoserine lactone and the formation of the virulence factors pyocyanin, cyanide, and lipase. Mol. Microbiol. 242, 309–319. (doi:10.1046/j.1365-2958.1997.3291701.x) 10.1046/j.1365-2958.1997.3291701.x 9159518

[36] Keen EC 2012 Paradigms of pathogenesis: targeting the mobile genetic elements of disease. Front. Cell. Infect. Microbiol. 2, 161. 23248780 10.3389/fcimb.2012.00161 PMC3522046

[37] Pirhonen M, Flego D, Heikinheimo R, Palva ET 1993 A small diffusible signal molecule is responsible for the global control of virulence and exoenzyme production in the plant pathogen *Erwinia carotovora*. EMBO J. 12, 2467–2476. (doi:10.1002/j.1460-2075.1993.tb05901.x) 8508772 10.1002/j.1460-2075.1993.tb05901.x PMC413482

[38] Guttman DS, Köhler T, Perron GG, Buckling A, van Delden C 2010 Quorum sensing inhibition selects for virulence and cooperation in *Pseudomonas aeruginosa*. PLoS Pathog. 6, e1000883 (doi:10.1371/journal.ppat.1000883) 20463812 10.1371/journal.ppat.1000883 PMC2865528

[39] Yu SC, Zhu XS, Zhou J, Cai ZH 2018 Data from: Biofilm inhibition and pathogenicity attenuation in bacteria by *Proteus mirabilis* Dryad Digital Repository. (doi:10.5061/dryad.sg4n7kb) 10.1098/rsos.170702 PMC5936886 29765621

